# Ultrasonography-Guided Lumbar Periradicular Injections for Unilateral Radicular Pain

**DOI:** 10.1155/2017/8784149

**Published:** 2017-03-30

**Authors:** Qing Wan, Shaoling Wu, Xiao Li, Caina Lin, Songjian Ke, Cuicui Liu, Wenjun Xin, Chao Ma

**Affiliations:** ^1^Pain Treatment Centre of Department of Rehabilitation Medicine, Sun Yat-sen Memorial Hospital, Sun Yat-sen University, Guangzhou, Guangdong Province, China; ^2^Department of Physiology and Pain Research Center, Zhongshan Medical School, Sun Yat-sen University, Guangzhou, Guangdong Province, China

## Abstract

*Objective*. The aim of this study was to compare the accuracy and efficacy of sonographically guided lumbar periradicular injections through in-plane or out-of-plane approach techniques for patients with unilateral lower lumbar radicular pain. The feasibility and accuracy of these techniques were studied by means of computed tomography (CT).* Methods*. A total of 46 patients with chronic unilateral lumbar radicular pain were recruited and randomly assigned to either the in-plane or out-of-plane injection group. A mixture of 3 mL 1% lidocaine and 7 mg betamethasone was injected. The visual analog scale (VAS) was used to assess pain before and after treatment.* Results*. The pain intensity, as measured by VAS, significantly decreased in both in-plane and out-of-plane injection groups.* Conclusions*. The sonographically guided periradicular injections are feasible and effective in treating lumbar unilateral radicular pain.

## 1. Introduction

Unilateral radicular pain is thought to be induced by inflammation or irritation of an exiting spinal nerve root originated from degeneration of intervertebral disc [1]. Nerve root blocking therapy is the most commonly performed minimally invasive management for low back pain. Steroids and local anesthetic are the most frequently used injectates [2, 3]. The underlying mechanism of steroid administration is to reduce inflammation by inhibiting release of proinflammatory mediators [4]. The nerve root blocking can be delivered by ultrasound-guided or fluoroscopy-controlled manner in clinical trials. Recently, the reliability and efficacy of ultrasound-guided injections in the lumbar spine have been broadly discussed and well accepted by patients and physicians because of the real-time guidance of injection and without radiation exposure [5–8].

With the real-time guidance of ultrasound, the spinous process and adjacent structures such as lamina, zygapophyseal articulations, and transverse process can by clearly identified. Several injection procedures have been introduced, including transforaminal injection through in-plane approach [9], medial branch block to the facet joint [10], and pararadicular injection through paramedian sagittal and paramedian sagittal oblique approaches [11]. The aim of our study is to compare the accuracy, safety, and the effect on pain relief of lumbar nerve root blocking through ultrasound guidance by in-plane and out-of-plane techniques.

## 2. Materials and Methods

### 2.1. Patient Characteristics

The study protocol was approved by the Institutional Review Board of Sun Yat-sen Memorial Hospital of Sun Yat-sen University, and written informed consent was obtained from all patients. There were 52 eligible patients with chronic unilateral lower lumbar radicular pain for more than 3 months and 46 patients participated in this randomized, single-blind study. The patients were recruited consecutively between January 2015 and September 2016. They were randomly assigned into two groups and received one sonographically periradicular injection through either in-plane approach (IP, *n* = 25) or out-of-plane approach (OP, *n* = 21) techniques, respectively. A mixture of 3 mL 1% lidocaine and 7 mg betamethasone was injected.

All patients were diagnosed for low back pain with unilateral radicular pain through clinical presentations, medical examinations, computed tomography (CT), or magnetic resonance imaging (MRI). The excluded criteria were systemic inflammatory disease, uncontrolled diabetes, infections, previous injections within 3 months, taking oral anti-inflammatory medication, receiving physical therapy or other injection therapy during this study, and having underwent surgery. The demographic data for patients were demonstrated in Table 1.

### 2.2. Ultrasound-Guided Periradicular Injections In-Plane Approach

**Figure 1:**
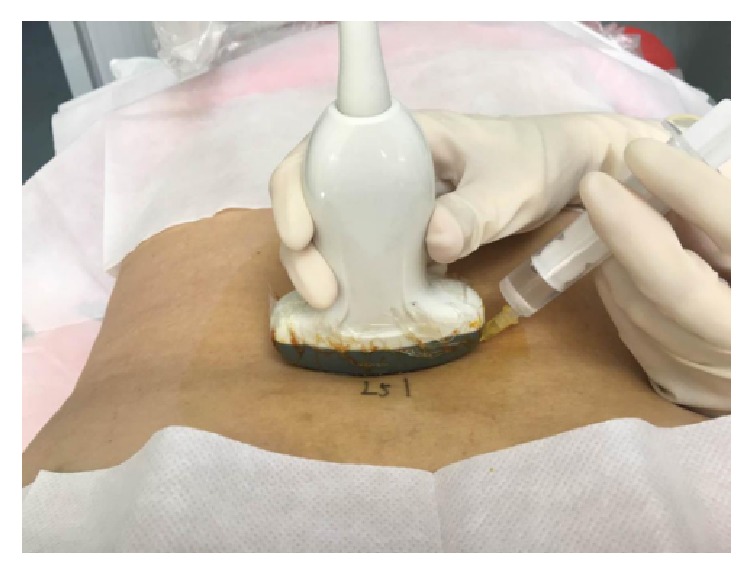
The position of the patient and the placement of transducer of in-plane approach.

**Figure 2:**
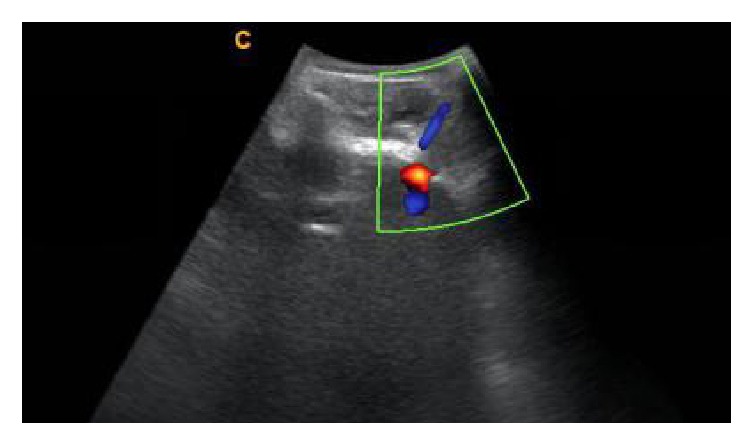
Transverse ultrasound image of the in-plane injection approach, the needle was inserted aiming to the Z-joint gap.

Ultrasound-guided selective nerve root block was conducted for 25 patients in 25 nerve roots. The patients were lying in the prone position with a pillow under the abdomen. The areas of injection treatment were disinfected and a sterile cover was placed on a curved array transducer. One experienced physician performed the ultrasound-guided injections using an Q9 (Xiang Sheng Company, Wuxi) device (Figure 1). The spinous processes were identified through a middle scan. First, the sacrum and the fifth lumbar spinous process were identified, and the target spinal level for the injection was confirmed by cephalad counting of the spinous process. At the target spinal level, a transverse axial plane was obtained by rotating the probe 90 degrees. The axial ultrasound image reflected the spinous process, lamina, facet joint, and transverse process. A needle (22 G) was inserted approximately 45 degrees into the skin using the in-plane technique, which enabled visualization of the path of the needle. When the needle tip reached the lateral side of the lamina or medial to the superior articular process (Figure 2), an inhalation test was performed to observe the presence of blood and cerebrospinal fluid. After confirming no inhalation, a mixture of 3 mL of 1% lidocaine and 7 mg betamethasone was injected.

### 2.3. Ultrasound-Guided Periradicular Injections Out-of-Plane Approach

**Figure 3:**
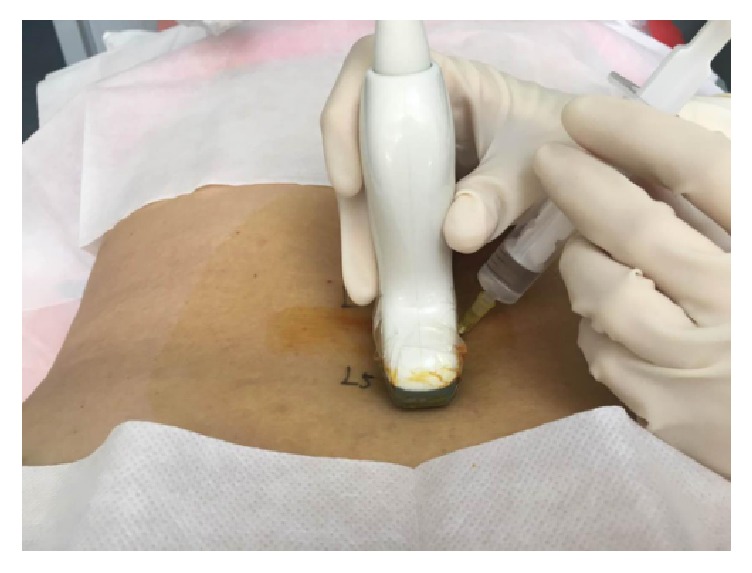
The position of the patient and the placement of transducer of out-of-plane approach.

**Figure 4:**
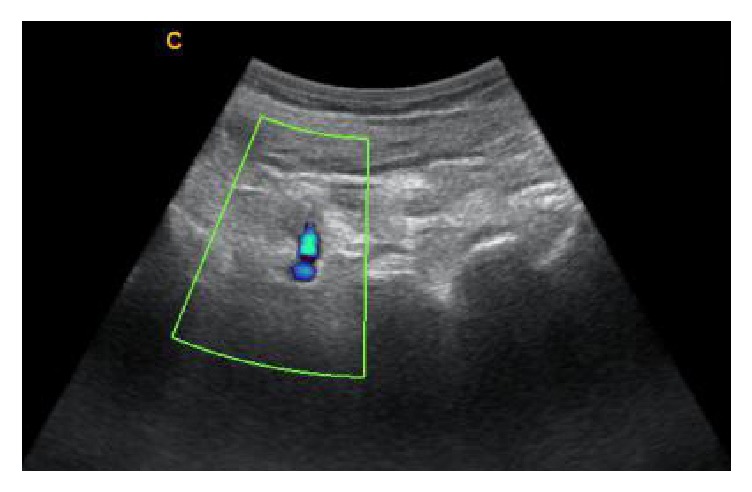
Longitudinal facet view was obtained and the needle was inserted approaching L4 nerve root.

The patients' position and sterilization were described previously. The transducer was placed longitudinally; the sacral spinous process and the fifth lumbar spinous process were identified. The lamina, facet joint, and transverse process were identified when moving the transducer from midline laterally. Then the transducer was moved back to visualize the edge of the zygapophyseal joints (Figure 3). After identification of the target injection level, a needle (22 G) was inserted approximately 70 degrees into the skin using the out-of-plane technique in the parasagittal view. The needle tip was located in the middle of the adjacent facet joints (Figure 4). The injection procedure and the medicine were described in the in-plane approach technique part.

### 2.4. Confirmation of Nerve Root Blocking by CT

**Figure 5:**
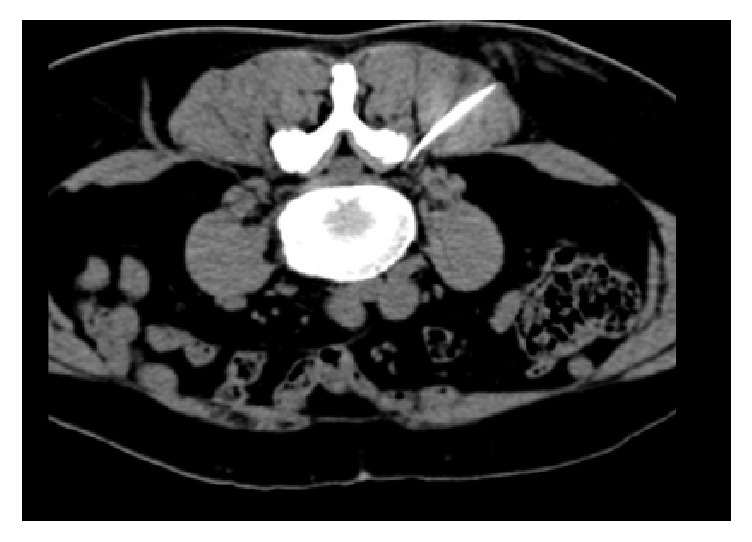
A representative image for confirmation of the needle pathway.

Patients were prepared as specified above for the US procedure. A radiopaque marker was placed at the indicated level. A low-dose topogram through the area of interest was obtained at 3 mm increments for a precise definition of the needle pathway by ultrasound guidance. A representative image for confirmation of the needle pathway is demonstrated in Figure 5.

### 2.5. Statistics

The data were analyzed using SPSS version 11.0 software (SPSS Inc., Chicago, IL, USA). Analysis of variance was used to compare the demographic characteristics of the patients, and a* t*-test was used for measurement data. *P* < 0.05 was considered statistically significant.

## 3. Results

### 3.1. Patient Characteristics

A total of 46 patients completed this study. The patients had comparable pain intensity assessed by VAS between IP (7.26 ± 1.00) and OP (7.34 ± 1.08) technique groups before injections. The spinal level of injections was located at L4 or L5. The demographic data of patients are present in Table 1.

### 3.2. Treatment Effects between the Approaches

The blocking procedures were tolerable for all the patients. None of the patients had any treatment-related complications.  Table 2 illustrates the procedure characteristics.

The pain was significantly reduced after injection in both IP and OP technique groups. There were no significant differences in the VAS before and after the injections between IP and OP technique groups (Table 2).

## 4. Discussion

Radicular low back pain is commonly caused by intervertebral disc herniation, spinal stenosis, and intervertebral disc degeneration. Selective nerve root blocking is one of the most frequently performed mini-invasive interventions. Ultrasonography has been used broadly in evaluation and treatment of musculoskeletal disorders. Ultrasound has proved to be reliable and accurate in the demonstration of paravertebral anatomy and sonographically guided lumbar injections for the treatment of unilateral lower lumbar radicular pain have been previously studied for feasibility and accuracy [12, 13]. The most challenging part in sonographically guided lumbar periradicular injections is in placing the needle in the exact position at the target nerve root. The advantage of parasagittal out-of-plane approach is deposition of medication close to the nerve root compared with in-plane approach aiming to the facet joint.

The success ratio of the lumbar nerve root blocking is over 95% in our study; this indicates that, in both approaches, the drug is able to be delivered at the periradicular space. Relief of symptoms has been the gold standard for evaluating the success of US-guided injection [14]. However, the symptoms could also be relieved by systemic drug effect even if the needle was not inserted at the precise position. The accuracy of ultrasound guidance, especially the needle tip, was evaluated by CT assessment.

In our study, there was no significant difference in VAS evaluation between IP and OP injection techniques. This indicates that, in both approaches, the medication is able to reach the periradicular space. The long-term pain relieving effect needs further investigation.

In comparison with CT, ultrasonography has several advantages. There is no radiation exposure for the physician. And the device is portable; it can be used in outpatient and at bedside. Despite the above advantages, it is has limitations in showing good quality view of spinal structures and the quality depends on experiences of the physician. It has been demonstrated that the reproducibility among physicians is low [15].

This study had some limitations: first, the sample size was small and the long-term effects were not evaluated; second, the outcome of injections was only pain score and functional tests of lumbar spine should be conducted in further researches.

## 5. Conclusions

The sonographically guided periradicular injections are feasible and effective in treating lumbar unilateral radicular pain.

## Figures and Tables

**Table 1:** Demographic data for patients.

Characteristic	IP technique (*n* = 25)	OP technique (*n* = 21)
Age, years (SD)	56.23 (10.30)	58.17 (9.62)
Sex M/F, *n*	16/9	13/8
Weight, kilograms (SD)	58.76 (8.31)	59.82 (7.20)
Height, meters (SD)	1.64 (0.05)	1.67 (0.04)
Body mass index, kg/m^2^ (SD)	21.70 (2.78)	21.14 (2.34)
Left/right, *n*	15/10	14/7
Spinal level of injection		
L4, *n* (%)	14 (56.0)	12 (57.1)
L5, *n* (%)	11 (44.0)	9 (42.9)
VAS (SD) before injection	7.26 (1.00)	7.34 (1.08)

**Table 2:** Procedure characteristics.

Characteristic	IP technique (*n* = 25)	OP technique (*n* = 21)	*P*
Patient treated, *n* (%)	23 (96.0)	20 (95.2)	0.567
Patient failed, *n* (%)	2 (4.0)	1 (4.8)	
Correct spinal segment identification, *n* (%)	24 (100)	20 (100)	
Accuracy of US- guided injection confirmed by CT, *n* (%)	22 (95.7)	19 (95.0)	0.720
VAS (SD) after injection	3.62 (0.81)	3.21 (0.90)	0.485
